# Impacts of climate change on fish hatchery productivity in Bangladesh: A critical review

**DOI:** 10.1016/j.heliyon.2022.e11951

**Published:** 2022-11-28

**Authors:** Mohammad Abu Baker Siddique, A.K. Shakur Ahammad, Abul Bashar, Neaz A. Hasan, Balaram Mahalder, Md. Mehedi Alam, Jatish Chnadra Biswas, Mohammad Mahfujul Haque

**Affiliations:** a Department of Fisheries Biology and Genetics, Bangladesh Agricultural University, Mymensingh, Bangladesh; b Department of Aquaculture, Bangladesh Agricultural University, Mymensingh, Bangladesh; c Department of Fishery Resources Conservation and Management, Khulna Agricultural University, Khulna, Bangladesh; d Krishi Gobeshona Foundation, BARC Complex, Farmgate, Dhaka, Bangladesh

**Keywords:** Fish hatchery, Climate change, Climatic variables, Broodfish, Seed or post-larvae, Aquaculture, Disease, Bangladesh

## Abstract

Bangladesh is among the countries most vulnerable to climate change due to its geographical location. Climate change issues have become major concerns in aquaculture industry, particularly for fish hatchery productivity. Fish production in Bangladesh is mainly steered by the aquaculture sector, which is dependent on private hatchery-based fish seed production to a great extent. This review aimed to present the impacts of climate change on fish hatcheries, particularly during different stages of hatchery production, and the economic loss from the onset of disease and other impairments due to environmental causes. Geographically, most hatcheries in Bangladesh are operated within a narrow range of temperature (22.8–23.1 °C, equivalent to 73–73.5 °F) and rainfall (1750–2000 mm). Thus, slightest fluctuations in these parameters affect seed production in fish hatcheries. The broodstock, produced in natural and captive conditions, is severely affected by flash flooding, water quality deterioration, river siltation, erratic rainfall, and temperature fluctuations. Based on our review, temperature fluctuation is the main factor hampering maturation and breeding performances of broodstock. Temperature has also been reported to affect embryonic development and cause stunted growth of larvae and juvenile. In shrimp and prawn hatcheries, fluctuations in temperature, pH, and salinity are responsible for post-larval disease outbreaks. In some instances, storms and heavy rainfall wash away reared broodfish and fish seed from the hatcheries, causing massive socioeconomic losses. This review presents indisputable negative impacts of climate change on hatchery production. As of now, no cost-effective proven strategies have been developed to minimize the effects of climate change on Bangladesh's fish hatchery production, on which the aquaculture industry is inextricably dependent. For sustainable fish hatchery production, basic research on climate impacts on hatcheries is inevitable, as well as improving capacity of hatchery owners are needed for resilient hatchery operations in Bangladesh and similar environments worldwide.

## 1 Introduction

Climate change is a global phenomenon considered as a major environmental issue in present time (Dutta et al., 2020). The major consequences of climate change include abrupt changes in temperature, erratic rainfall, and extreme climate events. Exacerbating the existing issues due to unusual seasonality and disease outbreaks, climate change is ravaging food production systems by sloping its productivity in harsh environmental conditions (Ray et al., 2019; Siddique et al., 2022). In the adverse impacts of climate change, fisheries and aquaculture are likely to be impacted in terms of ecosystems, biodiversity, breeding and productivity, and socioeconomic growth (Klinger et al., 2017). Although some studies argue that there are potential benefits of higher temperature in aquaculture production, but global warming has crossed the thermal limits of cultured species, causing heat stress in most of the tropical and sub-tropical countries (Froehlich et al., 2017). Climatic factors have a greater effect on fish physiology than in terrestrial animals and compared to terrestrial ecosystems, aquatic ones are more sensitive to environmental deviation. That is why the aquaculture industry is constantly being affected by the adverse effects of climate change.

Bangladesh is one of the global leading fish producing countries with a total production of approximately 4.5 million MT in 2019–2020, with fast growing aquaculture accounting for approximately 57.38% of the total production (Henriksson et al., 2014; DoF, 2020; Haque et al., 2021a; Haque et al., 2021b; Alam and Haque, 2021). Aquaculture in Bangladesh is considered as the sub-sector of fisheries (Bashar et al., 2022; Hasan et al., 2021; Heal et al., 2022) which was based on the stocking of wild seed; however, it is now almost (97.60%) replaced by hatchery-produced fingerlings (DoF, 2019). There are currently 963 privately-owned fish hatcheries in Bangladesh (DoF, 2020). Hatchery produced fish fry can be broadly divided into four categories such as carp (*Labeo rohita*, *Catla catla*, *Cirrhinus cirrhosus*, *Labeo calbasu*, *Labeo bata*, *Labeo gonius*, *Hypophthalmicthys molitrix*, *Aristichthys nobilis*, *Ctenopharyngodon idella*, *Cyprinus carpio*), catfish (*Pangasianodon hypophthalmus*, *Clarias batrachus*, *Heteropneoustes fossilis, Ompok pabda, Mystus cavasius*), tilapia (*Oreochromis niloticus*) and shellfish (*Penaeus monodon, Macrobrachium rosenbergii*). These species collectively contribute to approximately 80% of the total (2.58 million MT) aquaculture production in Bangladesh (DoF, 2020), indicating the importance of hatchery supplied seed production in the aquaculture of Bangladesh. Hatchery production of all the above mentioned fish species except *C. carpio* is mainly occurred from March to August i.e. during summer when the temperature ranges from 25 to 28 °C. The growing number of literature pertaining to the aquaculture producing countries and Bangladesh reports that fish hatchery production is extremely sensitive to climate change due to the skewed nature of production. The divergent climatic variability in Bangladesh is due to its unique geographic location, dominance of floodplains, low elevation from the sea, overwhelming dependence on nature, anthropogenic activities of the highly dense population, and high levels of poverty (Faruque and Kabir, 2016). Approximately 12% of Bangladesh's population are directly or indirectly associated with capture fisheries and aquaculture activities (DoF, 2022), and fish farming practices and associated livelihoods are vulnerable to the adverse impacts of climate change, with an amalgamation of changes in physical environments, aquatic ecosystems, farming operations, and diversified livelihood activities (Islam et al., 2020). This has caused a decline in wild seed production from natural sources and increased dependency on artificial seed production from hatcheries to operate aquaculture farms (Belton et al., 2011).

There are several stages of fry production in fish hatcheries in which rising temperature, to a certain level, influence gonadal maturation, ovulation, hatching, and larval development positively. Rainfall is a vital factor for the acceleration of sensational responses and hormonal functions of fish (Servili et al., 2020). In recent years, studies have shown that extreme temperature and rainfall events are adversely affecting the production of fish fry in hatcheries (Lebel et al., 2016). Moreover, extreme climatic events are pushing production back by damaging hatchery infrastructure and broodstock. To date, no established approaches have been developed to address the impacts of climate change on fish hatchery and aquaculture industry in Bangladesh. Some studies reported successful outcomes in fish farms and hatcheries despite the challenges of adopting cost-effective mitigation and adaptation approaches to climate change into practice. In this context, Alam et al. (2021) asserted that during months of high temperature, commercial fish hatcheries used sheds over the broodstock hapa (i.e., the rearing of broodfish in a closed net system in ponds) that produced more eggs. In Andhra Pradesh, Karnataka, Gujarat, Odisha, and West Bengal states of India, fish farmers used fresh water from nearby surface or sub-surface sources to lower water temperature, oxygen tablets to supplement oxygen during the summer, and nets in pond dikes to prevent fish from escaping during flooding (Subhendu et al., 2018). In the absence of evidence based mitigation strategies, climate change is disrupting fish seed production, jeopardizing the socioeconomic conditions of hatchery owners and operators. Depending on the literature available, no study has reviewed the wider impacts of climate change on the performance of fish hatcheries and their mitigation strategies in Bangladesh. In the interplay between climate challenge and hatchery industries, the main purpose of this work is to provide guidance on the impacts of climate change on hatchery productivity and the development of mitigation strategies.

## 2 Materials and methods

**Figure 1:**
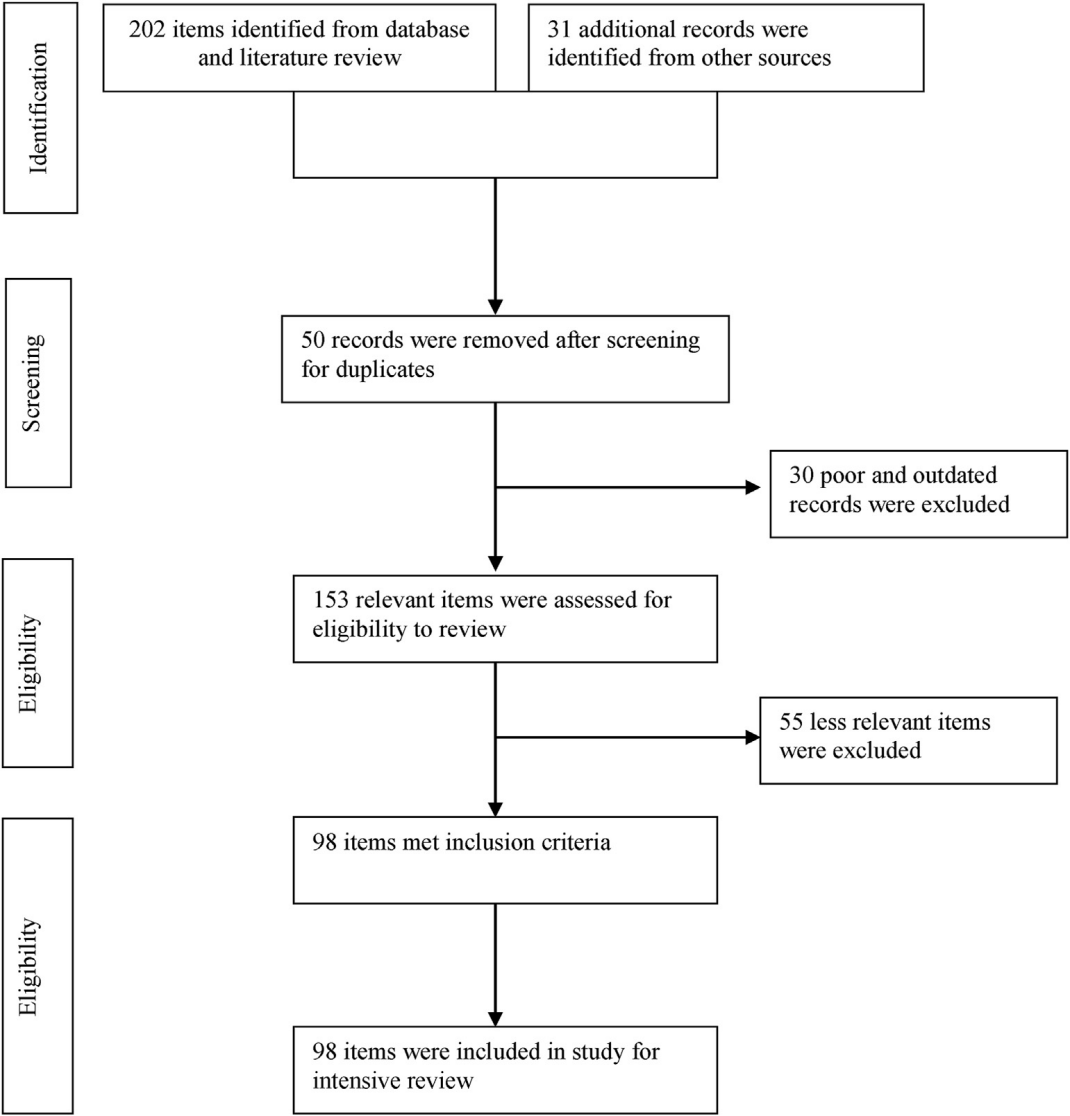
Screening literature using Preferred Reporting Items for Systematic Reviews and Meta-Analysis (PRISMA).

Focusing on the four types of fish species (carp, catfish, tilapia and shellfish) mentioned above, whose seed are produced at specific time of the year, this study was conducted. This study investigated the direct and indirect impacts of climate change at different stages of fish hatchery operations, such as broodstock rearing, breeding and spawning, larval development, disease occurrence, and economic losses in Bangladesh by reviewing and collecting data from various sources. We conducted a systematic review of literature based on the Web Science, Google Scholar, PubMed, and Scopus databases. Additional information was collected from government and non-government organizations and their databases, such as the Department of Fisheries (DoF), Bangladesh Fisheries Research Institute (BFRI), different agricultural and science and technology universities in Bangladesh, and open databases, particularly the digital repository of libraries of different universities (http://dspace.bau.edu.bd, https://www.library.juniv.edu/, http://library.bracu.ac.bd/, and https://www.griffith.edu.au/library). We adopted the Preferred Reporting Items for Systematic Reviews and Meta-Analysis (PRISMA) guidelines to analyze 98 reliable literatures related to the direct and indirect impacts of climate change on hatchery productivity of above mentioned fish species categories (Figure 1). In case of lack of information, literature on countries similar to Bangladesh in terms of their agro-ecological condition was also reviewed and compared. In many studies, PRISMA has been proven to improve the reporting quality of a systematic review and provide substantial transparency in the selection process of papers in a systematic review (Hoque et al., 2020; Hosen et al., 2021; Kundu et al., 2020). The key findings from the review of literature are summarized in Table 1. The spatial Global Positioning System (GPS) coordinates of hatchery clusters have been collected from the DoF database and spatially presented in relation to climatic zones of Bangladesh. The base map was generated in ArcGIS software by ESRI (ArcMap 10.8) using DIVA-GIS shape file.

**Table 1:** Impacts of climate change on different domains of fish hatchery operations concerning to productivity.

Domain of impacts	Species	Climatic variables	Specific impacts	References
Broodstock development	Carps	Flash flood and water quality deterioration	Huge mortality of broodfish in open-water	Deshwara (2017)
	Carps	Climate induced river siltation	Breeding ground destruction	Akhter and Rahman (2016)
	Indian major carps	Rainfall and change in flood regimes	Reduced seed production in hatcheries	Vass et al. (2009)
	Tilapia	Rising temperature	Occasions of egg collection from a tilapia brood have been reduced from 11 to 7 times	Haque (2009)
Breeding and spawning	Tilapia	Temperature	Poor spawning in both high and low temperature. Egg production declined at 33 °C and stopped at 35 °C	M. S. Hossan et al. (2013)
	Tilapia	Temperature	Growth performance of tilapia decreased after 34 °C and such tendency continued above this temperature	Rahman et al. (2021)
	Common carp	Temperature	Negatively impacted different stages of breeding	Usman et al. (2015)
Hatching and larval development	Tilapia	Temperature	Temperature affects hatching and development of larvae	Hossain et al., 2021
	Tilapia	Temperature	Increased water temperature (from 30 to 33 °C) significantly decreased larval development.	Faruk et al. (2012)
	Climbing perch	Temperature	Manipulated temperature stimulated growth-related gene expression at juvenile stage	Ahammad et al. (2021)
Disease occurrence	Shrimp	Temperature	Temperature fluctuations caused disease outbreak in hatcheries	Ahmed and Diana, 2015a, Ahmed and Diana, 2015b
	Prawn	Fluctuation of pH, salinity, and temperature	Caused viral diseases in prawn and shrimp	Arcier et al. (1999)
	Grass carp, Common carp	High temperature	Caused mainly viral and other microbial diseases	Cheng et al., 2008
Impacts on economy	Fish hatchery of carp and other species	Storms, temperature fluctuation and erratic rainfall	Caused massive economic losses through adverse effects on breeding, hatching and nursing	Bhattacharjya et al. (2022)
	Brood fish of carp and other species	Heavy rainfall	Washed away of reared brood fish from fish hatcheries due to heavy rainfall driven flood	Bhattacharjya et al. (2022)

The synthesized results of this article begin in Section 3 by presenting spatial distribution of fish hatcheries in different climatic zones of Bangladesh followed by Section 4 that introduces the different steps of hatchery operations being affected by climate change. Section 4 consists of the impacts of climate change on broodstock development, breeding and spawning, hatching and larval development and diseases occurrence. Section 5 presents the impacts of climate change on hatchery economy, then Section 6 concludes with recommendations.

## 3 Spatial distribution of fish hatcheries in different zones

**Figure 2:**
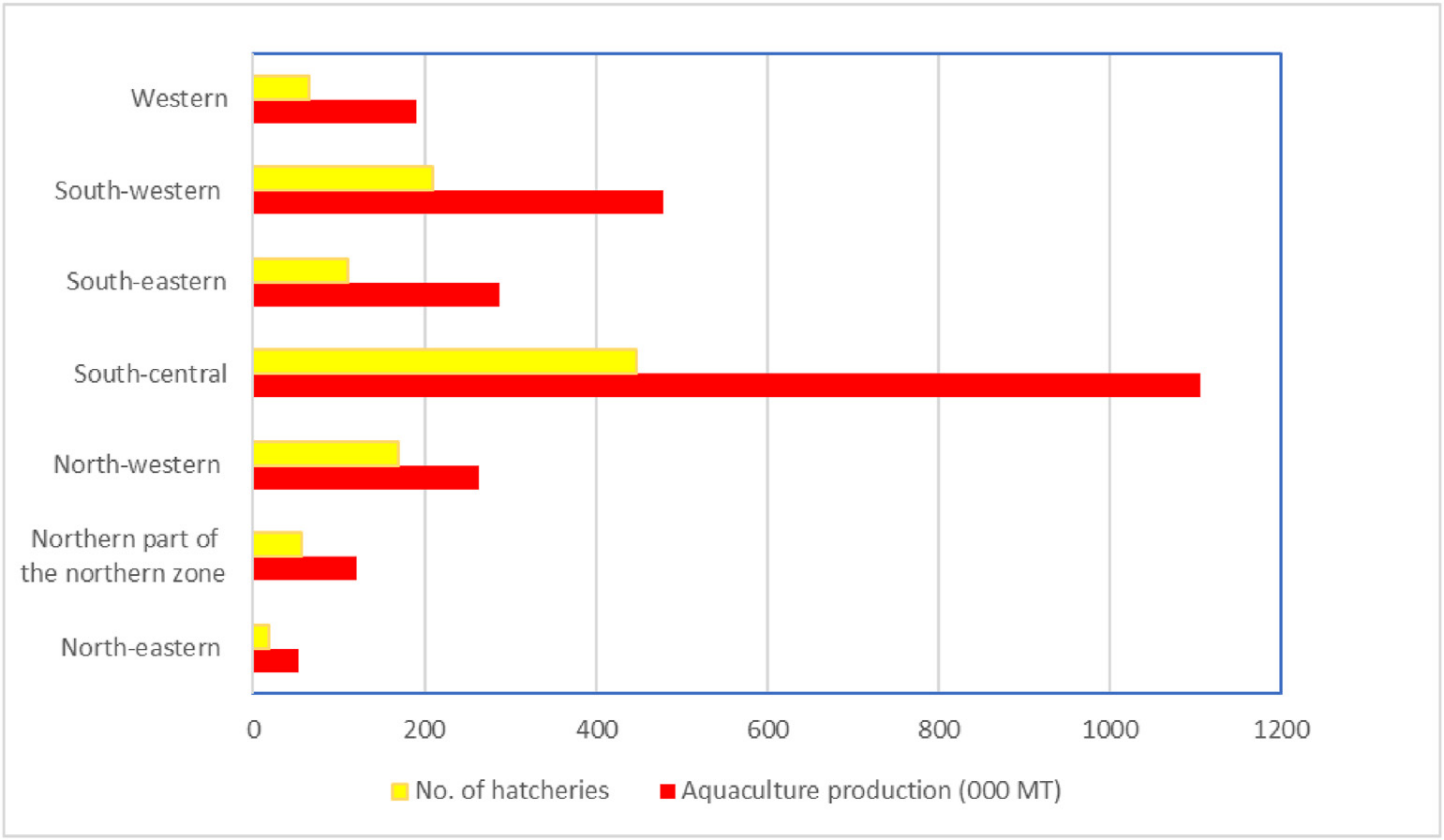
Relationship between aquaculture production and number of hatcheries in different climatic zones of Bangladesh.

The number of hatcheries in Bangladesh has increased significantly in the last two decades. The total number of government and private hatcheries in Bangladesh in 2001 was 112 and 671, respectively (DoF, 2003). In 2021 the number of government and private hatcheries stood at 103 and 963, respectively (DoF, 2021). These changes in number of hatcheries indicates that the number of government hatcheries in Bangladesh is rather decreasing, and the number of private hatcheries is increasing day by day on which the aquaculture of Bangladesh depends. Spatial analysis shows that the density of fish hatcheries is highest in Mymensingh and Jashore districts followed by Bogura and Cumilla districts. Figure 2 clearly indicates the strong (R^2^ = 0.97) relationship between the number of fish hatcheries and aquaculture production in the different climatic zones of Bangladesh. However, the Bogura district is an exception where hatchery productions are favored by climate condition, but grow-out system is in decline due to soil quality (red soil) (Talukder et al., 2018). Hatchery production was found dominating in certain districts in the country that have a distinct climatic tract, indicating subtle relationships between seed production and climatic variables. Climate change undoubtedly affects fish hatchery performance at an unprecedented rate in other parts of the world including the USA (Hanson and Ostrand, 2011). To predict these negative impacts, it is important to understand the extent to which fish hatcheries depend on climate factors.

**Figure 3:**
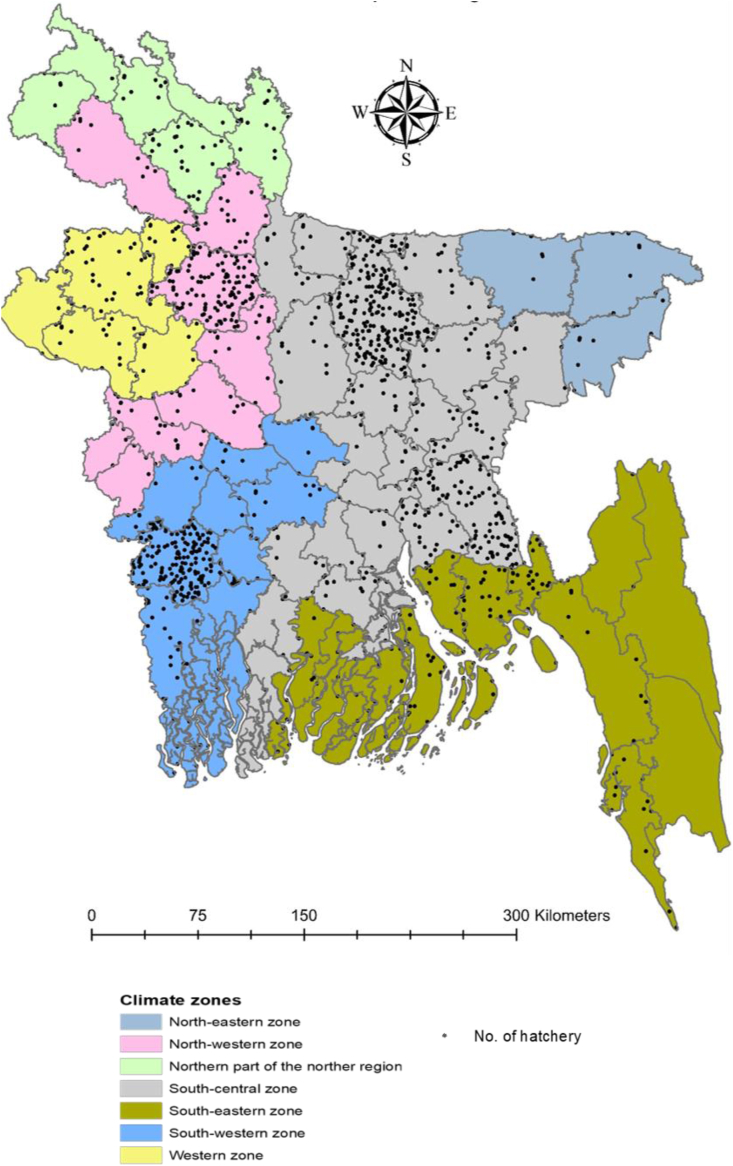
Geographical location of hatcheries in different climatic zones of Bangladesh.

The fish hatchery industry has bloomed in particular hubs of Bangladesh based on meteorological characteristics and water quality (for example, salinity, and pH). Among all climatic parameters, temperature and rainfall influence the breeding of fish sharply in both hatcheries and wild conditions (Meynecke et al., 2007). Bangladesh belongs to a sub-tropical zone; however, variations in rainfall, temperature, and humidity have created seven distinct climatic zones (Sarker et al., 2019). Fish hatchery production is dominant in the south-central zone, followed by the south-western and north-western zones (Figure 3). The south-central and north-western zones are characterized by average annual temperatures ranging between 22.8 °C and 23.1 °C and average annual rainfall between 1750 and 2000 mm (Rahman, 2018). The south-western zone has a lower temperature and rainfall range; however, the Jashore district, which harbors numerous hatcheries, experiences the highest temperature. From the map (Figure 3) showing the number of hatcheries in each district, we can identify three potential hatchery hotspots (Mymensingh, Bogura, and Jashore) in Bangladesh (Alam et al., 2019). Mymensingh and Bogura fall within the same latitude (25.00 N–24.30 N) where climatic variables remain identical. However, Jashore and aligned districts (at 23.00 N 23.50 N) like Cumilla niche a numerous hatcheries, but show less productivity and species diversity compared to that of Mymensingh and Bogura.

**Figure 4:**
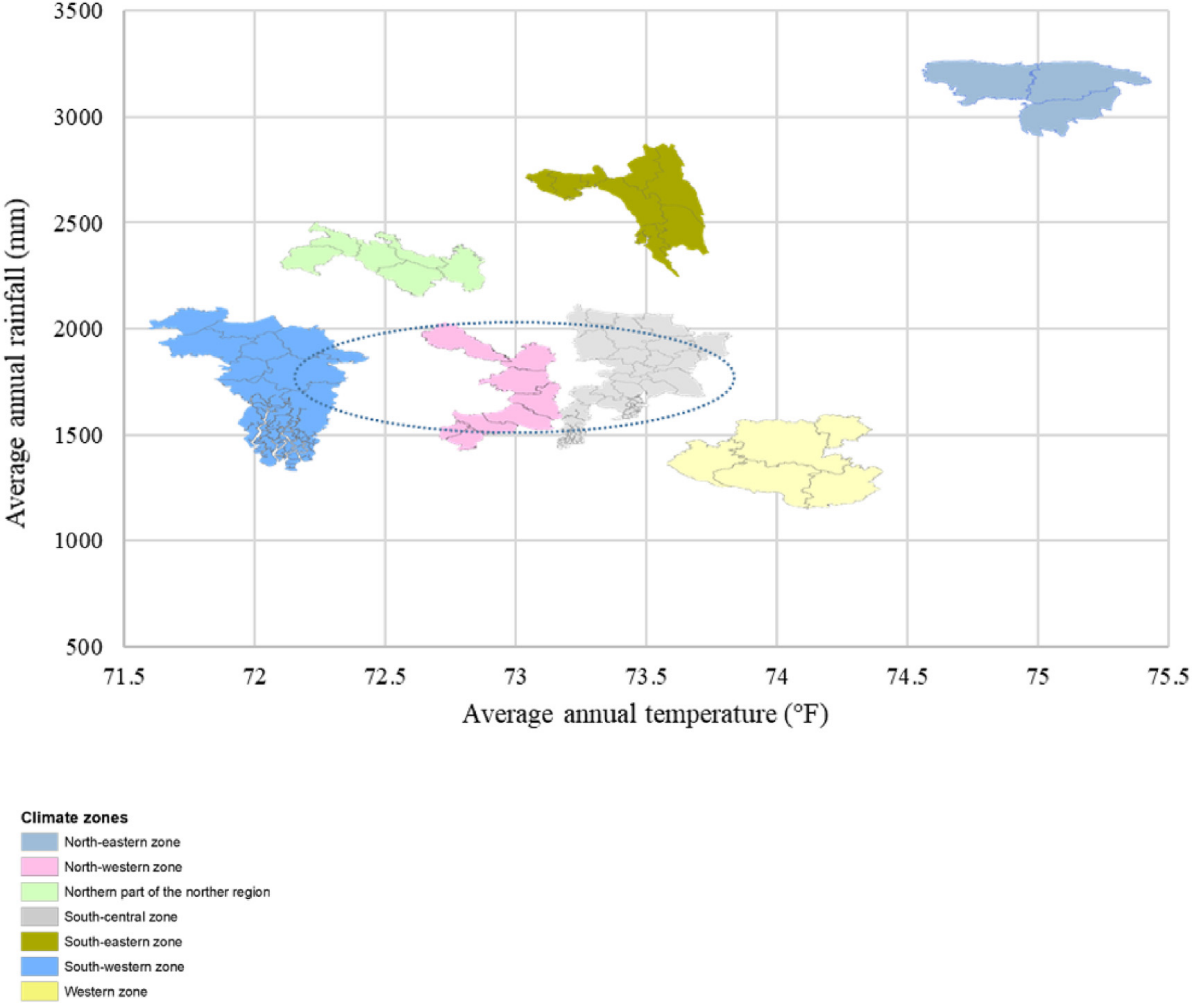
Annual average temperature and rainfall in different climatic zones of Bangladesh, showing the favorable range at which hatchery hubs are situated.

On observing the climate characteristics, it is evident that most of the hatcheries are established and operated in a narrow window of temperature (22.8 °C–23.1 °C) and rainfall (1750–2000 mm) (Figure 4). However, slight variation in temperature (e.g., 23.8 °C) may reduce the productivity of hatcheries in terms of seasonality, species diversity, and/or production intensity as seen in the south and north-western zones of Bangladesh. Climate change is predicted to translate into extreme temperature and erratic rainfall in the coming years; it is most likely to affect hatchery productivity, particularly in Bangladesh which is considered as the second most vulnerable country to climate change, and has been identified as one of the least adaptive countries in Asia.

## 4 Impact of climate change on hatchery productivity

Climate change refers to long-term shifts in weather pattern, and its major consequences include changes in weather variables such as temperature, rainfall, and extreme climatic events (e.g. cyclone, flood, drought, etc.). In this study we have included the impacts of both changes in weather variables and extreme climatic events, on fish hatchery operations. In other words, we have presented here the direct and indirect impacts of climate change that affected fish hatcheries (Table 1).

### 4.1 Impacts on broodstock development

Broodstock is usually collected from wild environment and then kept in ponds or tanks where stable temperature, pH, photoperiod, and other physicochemical parameters are maintained. Broodstock collected from the wild often undergo conditioning to ensure healthier stock and maximum productivity. Broodstock grown in open water or nurtured in closed water may be subjected to stressful environmental conditions and/or management practices seasonally or annually, thereby resulting in reduced reproductive performance. In April 2017, the early incessant rain caused a flash flood in Haor^1^ that further rendered inundation and decomposition of immature rice plants and grains, reducing the oxygen level in water and producing toxic gases, which eventually caused the death of a massive number of fish (Deshwara, 2017). Brood fish migrate from their winter habitat to breeding grounds at the end of February until the first week of March. Due to a lack of regular rainfall and scarcity of water in rivers and in the associated channels, the brood fish could not reach the breeding ground in time. According to Akhter and Rahman (2016), because of siltation in the river basin, two breeding grounds have been destroyed completely; one at the Kalikapur River in Noakhali, a breeding ground for Gonia fish; and another at the Bangali River in Bogura, a breeding ground for Rohu. Heavy rainfall in short duration due to climate change carried sand, boulder, and silt that filled up the migration routes in the Haor's channels and rivers, thereby hampering breeding migration and ultimately causing a decline in capture fisheries production (Aziz et al., 2021).

There is an increasing dependency of hatcheries on natural broodstock. For example, carp broodstock originating from Halda River is the most desirable germplasm to hatchery operators in Bangladesh. Changes in rainfall and flood regimes negatively impacted the breeding seasons of Indian major carps in their natural habitats, thereby reducing hatchery production (Vass et al., 2009). Since fish are poikilothermic, and weather in Bangladesh is highly seasonal, fish seed production and its demand play an important role for grow-out farmers. Since commercially farmed fish seed are produced in hatcheries at a specific time of the year during summer, seed production and availability influence aquaculture production to a greater extent. It has been reported that broodstock management is one of the most important aspects of tilapia seed production (Alam et al., 2021; Chaput et al., 2020). According to Haque (2009), hatchery-based broodstock development in the Mymensingh region (one of the most advanced aquaculture producing regions in Bangladesh) was reported to be affected severely by the rise in temperature. This study also found that tilapia hatcheries have not developed broodstock and did not produce seed as per their expected level in this region. For example, fertilized eggs from individual tilapia brood were collected 11 times in 2008, but only 7 times in 2009 (Haque, 2009). This variation in egg production might be related to prevailing rainfall and flooding regimes (Vass et al., 2009). Several studies have shown that a range of climatic (temperature, rainfall, sunlight) factors associated with nutritional (feed quality and quantity) and social (sex ratio, size structure, and dominance/hierarchy) factors play important roles in the synchronization of broodstock spawning (Migaud et al., 2013). For broodstock development in adverse climatic conditions, there is no mitigation strategy documented in literature for hatchery owner friendly practice in Bangladesh. According to Karmakar et al. (2018), genetic advancements, applying a variety of feed ingredients, superior formulation of feed, strict quality control of feed, and effective feeding strategies can contribute to broodstock development in adverse climate. Considering the above stated issues, research evaluating the reproductive performance of important cultured species, especially for adjustments in broodstock husbandry in the changing climatic conditions, needs to be conducted.

### 4.2 Impacts on breeding and spawning

Literature screened through PRISMA shows that fish breeding and spawning are hampered by the effects of climate change (Servili et al., 2020). High temperature and inadequate rainfall hampered gonadal development, breeding success, fertilization, embryonic development, and survival rate (Akhter and Rahman, 2016; Pankhurst and Munday, 2011). The average temperature increased by 1.4 °C between 1981 and 2010; and rainfall decreased by 25 mm between 1980 and 2008 in Tanguar Haor. These variations have negatively affected wild broodstock breeding and spawning that are commonly used in hatcheries (Rahaman et al., 2015; Rouf et al., 2011). Temperature change, abrupt rainfall patterns and extreme climatic events (cyclones, floods) affect the hatching rate of fish and yolk size of larvae (Munday et al., 2009), causing increased mortalities (Gagliano et al., 2007) because of osmotic stress (Islam et al., 2020). Brood fish breeding and spawning require optimum temperatures, thus seed production from different types of broods vary greatly. In Bangladesh, for example, the tilapia fish breeds between February and November when water temperature remains around 22–30 °C (Hussain, 2004). Following variable environmental conditions, female tilapia tend to spawn asynchronously every 3–4 weeks (Coward and Bromage, 2000; Macintosh and Little, 1995; Rana, 1988).

Considering the optimum growing temperature window, tilapia hatchery operations in Bangladesh start from February and continue until November for economic productivity (Alam et al., 2021). Tilapia do not produce eggs at temperatures below 19 °C (Brummett, 1995) and at 35 °C or higher (Hossain et al., 2013). A possible reason for this is that temperature in Bangladesh, during the May–September period, is likely to increase in the future (Basak et al., 2013). Moreover, based on latest literature, in recent years the maximum temperature during summer in Bangladesh remains between 30 °C and 40 °C (Alam et al., 2021). Due to this abrupt fluctuation of temperature during the transition of summer and winter, there are rarely any operating hatcheries during the winter breeding months which leads to shortage of tilapia seed during the winter season, affecting production. Growth performance of brood tilapia decreased at temperatures of 34 °C and higher. In contrast, the growth rate decreased at temperatures below 21 °C (Rahman et al., 2021). Moreover, the survival rate of brood tilapia at low temperatures is hampered. The reproductive performance in tilapia stays at a good level when temperature remains near or below 32 °C (Hossan et al., 2013). Male tilapia provided their best performance at temperatures between 28–32 °C (Rahman et al., 2021). There are no established adaptation strategies for adopting broodstock breeding and spawning in adverse climatic conditions at the hatchery level. In this connection, Subhendu et al. (2018) suggested a strategy which is a combination of artificial water flow and temperature control in hatchery system to stimulate broodfish breeding and spawning. Apart from climatic factors, broodstock breeding artificially depends largely on other management factors (Usman et al., 2015) which have to be studied in control and field conditions to understand the complex impacts of climate change on breeding and spawning.

### 4.3 Impacts on hatching and larval development

Larval and juvenile development in fish hatcheries, and other biological processes of fish are strongly affected by a composite set of meteorological and water quality and parameters (Belton et al., 2011; Hasan et al., 2022). It has been reported that temperature influences egg hatching and larval survival as larvae are usually more sensitive than juveniles and adults to climate variability (Ahammad et al., 2021). The influence of temperature stress persists throughout the life cycle of eggs and larvae that survived overcoming temperature impression. Moreover, it has also been reported that manipulation of water temperature at embryonic and juvenile stages stimulates growth-related gene expression which results in better growth of fish (Ahammad et al., 2021). In thermostatic experimental studies, it has been reported that temperature plays an important role on the larval development of tilapia, and the best hatching and larval development were observed at an optimum temperature range of 25–29 °C. Subsequently, an increase in water temperature (from 30–33 °C) significantly decreased the larval development of tilapia (Faruk et al., 2012). Several studies reported that physical deformity of larvae of tilapia occurs at temperatures above 34 °C (Islam and van Amstel, 2021; Rahman et al., 2021; Wang and Tsai, 2000). However, tilapia larvae reared under thermocycles (31 °C: 25 °C = day: night) demonstrated greater growth and displayed a more rhythmic correlation of digestive enzymes and genes with their mealtime than larvae reared at a constant temperature of 28 °C (Santo et al., 2020). Therefore, application of thermocycles in the larviculture of tilapia may be helpful to increase seed production during summer. However, there is a need to pursue further studies to understand the complex impacts of climatic variables on hatchery operation in tropical Bangladesh.

### 4.4 Impacts on disease occurrence in hatchery ranging from larva to broodfish

Disease is an indispensable incidence in all stages of aquaculture, starting from seed production to grow-out. Disease originating from viruses, true bacteria, Rickettsial-like bacteria, fungi, and protozoa are the main driving forces of economic losses in aquaculture production (Thornber et al., 2022), namely in finfish and shrimp hatcheries. Climate induced disease in hatcheries (both finfish and shrimp) are an unpopular and ignored research area in Bangladesh. However, each category of disease mentioned earlier, and their correspondent causative agents are impressionable to climatic factors. Considering this issue, diseases of finfish and shrimp hatcheries frequently mentioned by different researchers (see details in supplementary material: Tables 2 and 3) are discussed with the lens of agro-ecological factors of Bangladesh.

#### 4.4.1 Repercussion of finfish hatchery microbiomes toward environmental stress

Finfish hatcheries suffer from a wide range of microbial disease resulting in innumerable economic loss. Spring viremia of carp virus (SVCV) is the most documented pathogen in hatchery confinements, which shows its haughty nature (start to infect juveniles from 22 °C) in summer months (Ahne et al., 2002). The development of infections with grass carp reovirus (GCRV) (Fang et al., 1989; Ke et al., 1990), Koi herpesvirus (KHV) (Perelberg et al., 2008), Cyprinid herpesvirus-1 (CyHV-1) (MacLachlan and Dubovi, 2017), and Gill necrosis virus (Pikarsky et al., 2004) were reported to be due to high temperatures (25–30 °C for GCRV, 16–25 °C for KHV, <25 °C for CyHV-1, and 18–28 °C for Gill necrosis virus) and pH (3–10 for GCRV) that are a result of climatic conditions. In Bangladesh, the extended summer and increased temperature during winter months due to global warming (Islam et al., 2020; Kafy et al., 2021) are suspected to increase the amount of viral agents in finfish hatcheries. For some bacterial pathogen temperature fluctuation (20–30 °C for *Flexibacter columnaris*: Wakabayashi, 1991; 25–30 °C for *Edwardsiella ictaluri*: Nagai et al., 2008) acts as a predisposing factor for infection of the larval population.

Protozoans and parasites have been reported in the last decades as crucial climacteric infections having a substantial influence on economic loss for carp hatcheries and seed production (Mohan, 1999). Higher or rapidly fluctuating temperatures due to climate change increase infectivity, reproduction, and growth of parasites (Macnab and Barber, 2012) and protozoans (Blanco and Unniappan, 2022) resulting in production loss. Therefore, it is important to study how the existing and forthcoming climate change influences protozoan and parasitic disease dynamics in fish (Barber et al., 2016).

#### 4.4.2 Repercussion of shrimp hatchery microbiomes toward environmental stress

Shrimp hatcheries in Bangladesh are mostly proliferated in climate-vulnerable coastal areas, which are prone to seasonal variations (Ahmed and Diana, 2015a). *Macrobrachium rosenbergii* nodavirus (MrNV) and White Spot Syndrome Virus (WSSV) are the two most devastating diseases for prawn and shrimp farms, respectively induced from impetuous fluctuations in pH, salinity, and temperature (Arcier et al., 1999; Hasan et al., 2020; Hasan and Haque, 2020; Qian et al., 2003). In the environment of continued climate change, some other disease like *Macrobrachium* Hepatopancreatic Parvo-like Virus (MHPV) has become a great concern in recent years, specifically for shrimp hatcheries at early- and post-larval stages (Farook et al., 2019; Lightner et al., 1993; Spann et al., 1997). Other causative agents of viral disease were reported to appear in shrimp hatcheries, including *Macrobrachium* Muscle Virus (MMV) and Infectious Hypodermal and Haematopoietic Necrosis Virus (IHHNV) (Heal et al., 2021) which warrant future awareness for climate sensitive shrimp hatcheries of Bangladesh.

Bacterial disease is also a growing concern in shrimp hatcheries and has led to many hatchery owners going out of business. Out of all bacterial agents, *V. harveyi* is possibly more subversive for countries with a tropical climate like Bangladesh. The fearsome nature of a wide salinity range tolerability (Abraham and Palaniappan, 2004), possession of environmental stress (highly diverse and rapidly fluctuated environments) adaptive gene (Grimes et al., 2009; Montánchez and Kaberdin, 2020), and holding idiosyncratic disease development nature of Quorum sensing (for details see Bassler, 1999) in harsh environments facilitate adaptation of *V. harveyi* to tropical environments. The claim of *Vibrio* sp. dispersion from the incidence of global warming (Montánchez and Kaberdin, 2020) is a prognosis for the shrimp hatchery industry.

Some ciliated protozoan species (for example, *Vorticella* sp.) deserve special attention as they are responsible for disease in *P. monodon* (Chakraborti and Bandyapadhyay, 2011), while *P. monodon* from the Sundarbans areas is a potential brood source for Bangladesh shrimp hatcheries. Moreover, the fecal-oral transmission nature of protozoans (O'Rourke and Rosenbaum, 2015) and impressionable behavior of protozoan or fungal organisms to fluctuated climatic and environmental parameters exacerbates the problem (López-Téllez et al., 2009). Climatic factors (temperature, rainfall, declining salinity due to rainfall, etc.) coupled with ambient water quality deterioration (alkalinity, dissolved oxygen, hardness, etc.) collapse shrimp hatchery production in Bangladesh by predisposing larvae, post-larvae, and broodstock to disease (especially viral and bacterial). A recirculatory aquaculture system (RAS) is a suggested method to produce fry while keeping the hatchery free of pathogenic diseases caused by the negative effects of climate change (Thornber et al., 2020). According to FAO/NACA et al. (2012) new production technology, improved management practices, and a greater understanding of the genetic and physiological basis of immunity need to be brought under research investigation to adjust aquatic animal health in adverse climatic condition. In addition, precise antigen identification, more effective adjuvants, and improved vaccine administration need to be researched for developing effective vaccination to the fish towards reducing pharmaceuticals and antibiotic uses.

## 5 Impacts on the fish hatchery economy

In general, the climate-related economic risks in hatchery operations have received little attention and are underrepresented, although it is likely to be a key element for successful aquaculture operation (Uppanunchai et al., 2015). Available literature suggests that climate change in terms of temperature fluctuation and erratic rainfall caused significant economic losses to fish hatcheries through their adverse effects on breeding, hatching, and nursing (Bhattacharjya et al., 2022). Extreme temperatures negatively affect metabolism and physiological processes of fish fry, which instigate overly rapid development of fry, leading to malformations and death, and ultimately economic loss through disrupted seed production (Dey et al., 2007; Uppanunchai et al., 2015). Excessive rainfall and floods may bring along harmful substances, causing economic losses and deaths as well as inflicting damage to equipment, breeding, and electrical systems (Sriyasak et al., 2014). Bhattacharjya et al. (2022) noted that Indian hatchery operators have suffered from significant economic loss through washed away reared brood fish from fish hatcheries. Snakehead seed production technology has been adopted in Vietnam however, climate change has seriously impacted seed production, narrowing down the production scale and resulting in heavy losses or lack of capital for reproduction (Quyen et al., 2016). Erratic and low rainfall leads to prolonged droughts resulting in water unavailability, increased turbidity, and reduced oxygen level in nurseries and brood ponds (Islam et al., 2018a). To meet water scarcity during a dry period, groundwater is pumped to maintain pond waters, which increases the seed production costs in hatcheries and nurseries (Hossain et al., 2015). Furthermore, farmers' demand for fish seed has been greatly reduced due to the scarcity of water (either due to drying up earlier or inadequate water levels) for aquaculture. Das et al. (2011) noted that due to the drought in India, the price of fish seeds in various hatcheries reduced drastically and income declined by 61–73% in 2009. Cyclones may destroy hatchery infrastructure, roads, communications, and electricity transmission facilities, resulting in higher repair costs (Ahmed and Diana, 2015b; Biswas et al., 2019; Johnson and Hung, 2020). In 2021, Cyclone ‘Yass’ washed away a huge number of fish farms and hatcheries in Khulna, causing economic losses of approximately US$ 1.0 million (Subhani et al., 2021). Like other hatcheries, shrimp/prawn hatcheries are at high risk for economic losses due to climatic hazards in Bangladesh (Ahmed, 2011). About 76 shrimp/prawn hatcheries have been established in southwest coastal Bangladesh (DoF, 2020). All these enterprises are threatened by climatic factors and extreme climatic events such as coastal flooding, cyclones, heavy rainfall, and extreme sea surface temperature. Earlier studies have focused on how sea-level rise poses a great threat to shrimp/prawn hatchery operations (Ahmed and Diana, 2015b; Hossain et al., 2013) because of increased disease outbreaks (Islam et al., 2018; Lebel et al., 2018; Rahman et al., 2021). Subhani et al. (2021) reported that shrimp hatcheries in Cox's Bazar and Satkhira have suffered losses of around US$ 11.6 million due to bacterial disease from sea water intrusion. Despite the high demand for hatcheries-reared PL (post-larvae), climatic events have exacerbated the problems of hatchery operation along with other causes; thus, most hatcheries were reported to be less productive (Islam et al., 2018a). A multidisciplinary components, ranging from aquatic to terrestrial systems are associated with fish hatchery production, making it complex in mitigating losses due to climate change from an economic point of view. Karmakar et al., (2018) proposed a necessary adaptation procedure with a sustainable approach which is the development of community capacity and the ecosystem approach to resource management. To reduce the potential negative effects of adverse climate, people, communities, NGOs, governments, and policy-making agencies can all play a significant role. Fish hatcheries may have a 'hatchery-climate change action plan' to quickly adjust operations in case of unforeseen circumstances and to avoid future crisis situations due to climate change (De Silva and Soto, 2009). Hatchery owners need financial and insurance support from public or private initiatives to recover from hatchery losses due to extreme climatic events. FAO/NACA et al., (2012) proposed that destructions related to hatchery production or infrastructure due to adverse climatic events need to be covered by insurance facilities for keeping the flow of production in a sustainable manner.

## 6 Conclusion

Literature screened through PRISMA shows that the effects of climate on hatchery production in Bangladesh is subsisted, but unfairly avoided to bring to the frontline. In hatcheries, fish fry are produced through a series of successful interlinked operations. This study has shown that both finfish and shellfish hatcheries are adversely affected by the impacts of climate change at different stages of hatchery operation. The majority of the research works focused on the effects of temperature fluctuations and erratic rainfall combined with other climatic variables on fish hatcheries. The most significantly reported element that adversely impacted the broodstock development, breeding and spawning, hatching and larval development, and the incidence of fish diseases, is temperature fluctuation. Consequently the productivity of the hatchery decreases, and the hatchery owners face economic loss. Although some studies show some strategies for climate change adaptation and resilience to reduce the economic losses of hatchery owners, no such strategies have yet been established for sustainable hatchery operation in the changing climate. In this context, laboratory and field based action research with the hatchery owners might be undertaken to gain a better knowledge of how to maintain fish hatchery productivity in Bangladesh.

## Declarations

### Author contribution statement

All authors listed have significantly contributed to the development and the writing of this article.

### Funding statement

A. K. Shakur Ahammad was supported by Krishi Gobeshona Foundation (KGF) [CRP-II (Second Phase)].

### Data availability statement

Data will be made available on request.

### Declaration of interest’s statement

The authors declare no conflict of interest.

### Additional information

Supplementary content related to this article has been published online at [URL].

## Appendix A Supplementary data

Supplementary Materialv1 Supplementary Materialv1

## Footnotes

1 Haor is a saucer-shaped, low-lying depression on a floodplain or river swamp, part of which dries up during the dry season.
