# Supplementary material for: Impacts of climate change on fish hatchery productivity in Bangladesh: A critical review
Source: Heliyon. 2022 Nov 28;8(12):e11951. doi: 10.1016/j.heliyon.2022.e11951 (PMC9732313; doi:10.1016/j.heliyon.2022.e11951)
Supplement: Supplementary Materialv1 [file mmc1.doc]

Table 1. Major disease burden in shrimp hatcheries of Bangladesh

| **Disease name** | **Causative agent** | **Susceptible species** | **References** |
| --- | --- | --- | --- |
| White body disease^¥^ | *Macrobrachium rosenbergii* nodavirus (MrNV) | *M. rosenbergii* | Chen et al., 2021 |
| White spot disease^¥^ | White Spot Syndrome Virus (WSSV) | A wide range of species | Pradeep et al., 2012 |
| Macrobrachium muscle disease^¥^ | Macrobrachium Muscle Virus (MMV) | *M. rosenbergii* | Tung et al., 1999 |
| Hepatopancreatic parvovirus disease^¥^ | Macrobrachium Hepatopancreatic Parvolike Virus (MHPV) | A wide range of species | Catap et al., 2003; Safeena et al., 2012 |
| Infectious Hypodermal and Haematopoietic Necrosis (IHHN) ^¥^ | IHHN Virus | A wide range of species | Escobedo-Bonilla, 2011 |
| Luminescent bacterial disease^β^ | *Vibrio harveyi* | *Penaeus monodon* | Chrisolite et al., 2008 |
| Mid cycle disease^β^ | *Enterobacter aerogenes* | *M. rosenbergii* | Brock, 1988 |
| Necrotizing Hepatopancreatitis (NHP)^β^ | NHP bacterium | A wide range of species | Frelier et al., 1993; Vincent and Lotz, 2007 |
| Protozoan disease | *Zoothamnium* sp., *Epistylis* sp., *Vorticella* sp. | A wide range of species. *Peneaus monodon* is the potential host. | Chakraborti and Bandyapadhyay, 2011 |
| Fungal Diseases | *Lagenidium* sp., *Fusarium* sp.,  *Sirolpidium* sp. and *Saprolegnia* sp. | A wide range of species | Khoa et al., 2004; Vilela et al., 2015 |

^¥^ Viral disease; ^β^ Bacterial disease

Table 2. Major disease burden in finfish hatcheries of Bangladesh

| **Disease name** | **Causative agent** | **Susceptible species** | **References** |
| --- | --- | --- | --- |
| Grass carp haemorrhage disease^¥^ | Grass carp reovirus (GCRV) | Chinese carp | He et al., 2017; Zeng et al., 2016 |
| Koi herpesvirus disease^¥^ | Koi herpesvirus (KHV) | Carps, Koi | Ababneh et al., 2020; Bretzinger et al., 1999 |
| Spring viremia of carp (SVC)^¥^ | SVC virus | *Cyprinus carpio*, Other cyprinid fish | Lakshmi et al., 2019 |
| Carp pox^¥^ | Cyprinid herpesvirus 1 (CyHV-1) | *Cyprinus carpio*, Other cyprinid fish | Rahmati-Holasoo et al., 2020 |
| Gill necrosis^¥^ | Gill necrosis virus | *Cyprinus carpio* | Pikarsky et al., 2004 |
| Swim-bladder inflammation^¥β^ | *Pseudomonas* sp., *Aeromonas* sp., *Micrococcus* sp., *Rhabdovirus carpio* | *Cyprinus carpio*, *Catla catla* | Bachmann and Ahne, 1973; Jhingran and Pullin, 1985; Sirri et al., 2020, 2016 |
| Ulcerative diseases^β^ | *Aeromonas* sp., *Pseudomonas* sp., *Vibrios* sp., *Flavobacterium* sp. | A wide range of species | Califano et al., 2017; Loch and Faisal, 2014; Yazid et al., 2021; Öztürk et al., 2007 |
| Bacterial haemorrhagicsepticaemia^β^ | *Aeromonas hydrophila* | Carp | Öztürk et al., 2007 |
| Columnaris disease^β^ | *Flexibacter columnaris* | A wide range of species | Bernardet, 1989; Declercq et al., 2013; Leadbetter, 1974 |
| Dropsy^β^ | *Aeromonas hydrophila* | Indian major carp | Faruk and Anka, 2017 |
| Bacillary necrosis of *Pangasius* (BNP)^β^ | *Edwardsiella ictaluri* | *Pangasius* sp. | Faruk and Anka, 2017 |
| Carp erythrodermatitis^β^ | *Aeromonas hydrophila, Pseudomonas* sp. | *Cyprinus carpio* | Sioutas et al., 1991 |
| Haemorrhagic septicaemia^β£ƿ^ | *Aeromonas* sp., *Pseudomonas* sp., *Vibrios* sp., *Saprolegnia* sp. | *Cyprinus carpio*, *Labeo rohita* | Kousar et al., 2020 |
| Cotton wool disease^£^ | *Saprolegnia* sp. | Indian major carp, exotic carp | Faruk and Anka, 2017 |
| Gill rot^£^ | *Branchiomycess anguinis, B. demigrans* | A wide range of species | Faruk and Anka, 2017 |
| Epizootic Ulcerative Syndrome (EUS)^£^ | *Aphanomyces invadans* | A wide range of species | Kar, 2016 |
| White spot disease^ƿ^ | *Ichthyopthirius multifiliis* | *Catla catla*, *Labeo rohita*, *Cirrhinus mrigala* | Gardinassi et al., 2021 |
| Costiasis^ƿ^ | *Ichthyobodo* sp. | A wide range of species | Faruk and Anka, 2017 |
| Trichodiniais^ƿ^ | *Trichodinids* sp. | Tilapia and a wide range of species | Valladão et al., 2016 |
| Cryptobiosis^ƿ^ | *Cryptobia*sp. | A wide range of species | Palmeiro and Roberts, 2013 |
| Gill mixoboliasis^ϼ^ | *Myxobolus* sp., *Henneguya* sp. | Indian major carp | Jhingran and Pullin, 1985; Kaur and Ahmad, 2017 |
| Black spot disease^ϼ^ | *Diplostomum* sp. | *Catla catla*, *Labeo rohita*, *Cirrhinus mrigala*, *Hypophthalmichthys molitrix* | Dezfuli et al., 2021 |
| Argulosis^ϼ^ | *Argulus* | A wide range of species | Ahmed, 2004; Mohan, 2007 |
| Lernaeosis^ϼ^ | *Lernae* sp. | A wide range of species | Faruk and Anka, 2017 |
| Dactylogyrosis^ϼ^ | *Dactylogyrus* sp. | Tilapia and a wide range of species | El-Sayed, 2020 |
| Gyrodactylosis^ϼ^ | *Gyrodactylus* sp. | Tilapia and a wide range of species | El-Sayed, 2020 |
| Algal toxicosis disease^€^ | *Microcystis* sp., *Anabaena* sp. | Indian major carp, exotic carp | Faruk and Anka, 2017 |
| Gas bubble disease^€^ | Environmental factors (supersaturation of oxygen and nitrogen gas or extensive load of organic fertilizers) | Carp | Faruk and Anka, 2017 |

^¥^ Viral disease; ^β^ Bacterial disease, ^£^ Fungal disease, ^ƿ^ Protozoan disease, ^ϼ^ Parasitic disease, ^€^ Environmental disease

**References**

Ababneh, M., Hananeh, W., Alzghoul, M., 2020. Mass mortality associated with koi herpesvirus in common carp in Iraq. Heliyon 6, e04827. <https://doi.org/10.1016/J.HELIYON.2020.E04827>

Ahmed, A.T.A., 2004. Development of environment friendly medicant for the treatment of argulosis in carp brood stock pond. SUFER-DFID Final Report.

Bachmann, P.A., Ahne, W., 1973. Isolation and Characterization of Agent causing Swim Bladder Inflammation in Carp. Nature 244, 235–237. <https://doi.org/10.1038/244235a0>

Bernardet, J.F., 1989. ’Flexibacter columnaris’- first description in France and comparison with bacterial strains from other origins. Dis. Aquat. Organ. 6, 37–44. <https://doi.org/10.3354/DAO006037>

Bretzinger, A., Fischer-Scherl, T., Oumouma, M., Hoffmann, R., Truyen, U., 1999. Mass mortalities in Koi, *Cyprinus carpio*, associated with gill and skin disease. Bull. Eur. Assoc. Fish Pathol. 19, 182–185.

Brock, J.A., 1988. Larval Mid-cycle disease of *M. rosenbergii*, in: Sindermann, C.J., Lightner, D. V. (Eds.), Disease Diagnosis and Control in North American Aquaculture. Developments in Aquaculture and Fisheries Science 17. Elsevier, Amsterdam, pp. 137–139.

Califano, G., Castanho, S., Soares, F., Ribeiro, L., Cox, C.J., Mata, L., Costa, R., 2017. Molecular taxonomic profiling of bacterial communities in a gilthead seabream (*Sparus aurata*) hatchery. Front. Microbiol. 8, 204. <https://doi.org/10.3389/FMICB.2017.00204/BIBTEX>

Catap, E.S., Lavilla-Pitogo, C.R., Maeno, Y., Traviña, R.D., 2003. Occurrence, histopathology and experimental transmission of hepatopancreatic parvovirus infection in Penaeus monodon postlarvae. Dis. Aquat. Organ. 57, 11–17. <https://doi.org/10.3354/DAO057011>

Chakraborti, J., Bandyapadhyay, P.K., 2011. Seasonal incidence of protozoan parasites of the black tiger shrimp (*Penaeus monodon*) of Sundarbans, West Bengal, India. J. Parasit. Dis. 35, 61–65. <https://doi.org/10.1007/S12639-011-0032-9/FIGURES/9>

Chen, K.F., Tan, W.S., Ong, L.K., Zainal Abidin, S.A., Othman, I., Tey, B.T., Lee, R.F.S., 2021. The *Macrobrachium rosenbergii* nodavirus: a detailed review of structure, infectivity, host immunity, diagnosis and prevention. Rev. Aquac. 13, 2117–2141. <https://doi.org/10.1111/RAQ.12562>

Chrisolite, B., Thiyagarajan, S., Alavandi, S. V., Abhilash, E.C., Kalaimani, N., Vijayan, K.K., Santiago, T.C., 2008. Distribution of luminescent Vibrio harveyi and their bacteriophages in a commercial shrimp hatchery in South India. Aquaculture 275, 13–19. <https://doi.org/10.1016/J.AQUACULTURE.2007.12.016>

Declercq, A.M., Haesebrouck, F., Van Den Broeck, W., Bossier, P., Decostere, A., 2013. Columnaris disease in fish: a review with emphasis on bacterium-host interactions. Vet. Res. 44, 1–17. <https://doi.org/10.1186/1297-9716-44-27>

Dezfuli, B.S., Giari, L., Bosi, G., 2021. Survival of metazoan parasites in fish: Putting into context the protective immune responses of teleost fish, in: Rollinson, D., Stothard, R. (Eds.), Advances in Parasitology. Academic Press, Elsevier, pp. 77–132. <https://doi.org/10.1016/BS.APAR.2021.03.001>

El-Sayed, A.-F.M., 2020. Stress and diseases, in: Tilapia Culture. Academic Press, Elsevier, pp. 205–243. <https://doi.org/10.1016/B978-0-12-816509-6.00009-4>

Escobedo-Bonilla, C.M., 2011. Application of RNA Interference (RNAi) against Viral Infections in Shrimp: A Review. J. Antivir. Antiretrovir. S9, 1–12. <https://doi.org/10.4172/jaa.S9-001>

Faruk, M.A.R., Anka, I.Z., 2017. An overview of diseases in fish hatcheries and nurseries. Fundam. Appl. Agric. 2, 311–316. <https://doi.org/10.5455/faa.277539>

Frelier, P.F., Loy, J.K., Kruppenbach, B., 1993. Transmission of Necrotizing Hepatopancreatitis in *Penaeus vannamei*. J. Invertebr. Pathol. 61, 44–48. l<https://doi.org/10.1006/JIPA.1993.1008>

Gardinassi, L.G., Maruyama, S.R., Cantacessi, C., 2021. Editorial: Systems Biology of Hosts, Parasites and Vectors. Front. Cell. Infect. Microbiol. 11, 796475. <https://doi.org/10.3389/FCIMB.2021.796475/BIBTEX>

He, L., Zhang, A., Pei, Y., Chu, P., Li, Y., Huang, R., Liao, L., Zhu, Z., Wang, Y., 2017. Differences in responses of grass carp to different types of grass carp reovirus (GCRV) and the mechanism of hemorrhage revealed by transcriptome sequencing. BMC Genomics 18, 1–15. <https://doi.org/10.1186/S12864-017-3824-1/FIGURES/8>

Jhingran, V.G., Pullin, R.S. V., 1985. A Hatchery Manual for the Common, Chinese and Indian Major Carps. ICLARM Studies and Reviews 11, Asian Development Bank, Manila, Philippines and International Center for Living Aquatic Resources Management, Manila, Philippines.

Kar, D., 2016. Aspects of Investigation of Epizootic Ulcerative Syndrome Outbreaks, in: Kar, D. (Ed.), Epizootic Ulcerative Fish Disease Syndrome. Academic Press, Elsevier, pp. 95–176. <https://doi.org/10.1016/B978-0-12-802504-8.00006-7>

Kaur, H., Ahmad, I., 2017. A report on two new myxozoan parasites infecting gills of fingerlings of Indian major carps cultured in nursery ponds in Punjab (India). J. Parasit. Dis. 41, 987–996. <https://doi.org/10.1007/S12639-017-0923-5/TABLES/6>

Khoa, L. V., Hatai, K., Aoki, T., 2004. Fusarium incarnatum isolated from black tiger shrimp, *Penaeus monodon* Fabricius, with black gill disease cultured in Vietnam. J. Fish Dis. 27, 507–515. <https://doi.org/10.1111/J.1365-2761.2004.00562.X>

Kousar, R., Shafi, N., Andleeb, S., Ali, N.M., Akhtar, T., Khalid, S., 2020. Assessment and incidence of fish associated bacterial pathogens at hatcheries of Azad Kashmir, Pakistan. Brazilian J. Biol. 80, 607–614. <https://doi.org/10.1590/1519-6984.217435>

Lakshmi, B., Syed, S., Buddolla, V., 2019. Current Advances in the Protection of Viral Diseases in Aquaculture with Special Reference to Vaccination, in: Buddolla, V. (Ed.), Recent Developments in Applied Microbiology and Biochemistry. Academic Press, Elsevier, pp. 127–146. <https://doi.org/10.1016/B978-0-12-816328-3.00010-6>

Leadbetter, E.R., 1974. Order II: Cytophagales Nomen novum, in: Buchanan, R.E., Gibbons, N.E. (Eds.), Bergey’s Manual of Determinative Bacteriology. Williams and Wilkins, Baltimore, MD, pp. 99–122.

Loch, T.P., Faisal, M., 2014. Chryseobacterium aahli sp. nov., isolated from lake trout (*Salvelinus namaycush*) and brown trout (*Salmo trutta*), and emended descriptions of chryseobacterium ginsenosidimutans and chryseobacterium gregarium. Int. J. Syst. Evol. Microbiol. 64, 1573–1579. <https://doi.org/10.1099/IJS.0.052373-0/CITE/REFWORKS>

Mohan, C. V., 2007. Seed quality in freshwater fish production, in: Bondad-Reantaso, M.G. (Ed.), Assessment of Freshwater Fish Seed Resources for Sustainable Aquaculture. FAO Fisheries Technical Paper. No. 501. Rome, Italy, FAO, pp. 499–517.

Öztürk, D., Adanir, R., Turutoglu, H., 2007. Isolation and antibiotic susceptibility of *Aeromonas hydrophila* in a carp (*Cyprinus carpio*) hatchery farm. Bull. Vet. Inst. Pulawy 51, 361–364.

Palmeiro, B.S., Roberts, H.E., 2013. Gastrointestinal Protozoal Parasites, in: Mayer, J., Donnelly, T.M. (Eds.), Clinical Veterinary Advisor: Birds and Exotic Pets. W.B. Saunders, Elsevier, pp. 28–29. <https://doi.org/10.1016/B978-1-4160-3969-3.00019-6>

Pikarsky, E., Ronen, A., Abramowitz, J., Levavi-Sivan, B., Hutoran, M., Shapira, Y., Steinitz, M., Perelberg, A., Soffer, D., Kotler, M., 2004. Pathogenesis of Acute Viral Disease Induced in Fish by Carp Interstitial Nephritis and Gill Necrosis Virus. J. Virol. 78, 9544–9551. <https://doi.org/10.1128/JVI.78.17.9544-9551.2004/ASSET/236C67CC-BCAE-422A-AC1A-B2ECCEA96126/ASSETS/GRAPHIC/ZJV0170450400008.JPEG>

Pradeep, B., Rai, P., Mohan, S.A., Shekhar, M.S., Karunasagar, I., 2012. Biology, Host Range, Pathogenesis and Diagnosis of White spot syndrome virus. Indian J. Virol. 23, 161–174. <https://doi.org/10.1007/S13337-012-0079-Y>

Rahmati-Holasoo, H., Ahmadivand, S., Shokrpoor, S., El-Matbouli, M., 2020. Detection of Carp pox virus (CyHV-1) from koi (*Cyprinus carpio* L.) in Iran; clinico-pathological and molecular characterization. Mol. Cell. Probes 54, 101668. <https://doi.org/10.1016/J.MCP.2020.101668>

Safeena, M.P., Rai, P., Karunasagar, I., 2012. Molecular biology and epidemiology of hepatopancreatic parvovirus of penaeid shrimp. Indian J. Virol. 23, 191–202. <https://doi.org/10.1007/S13337-012-0080-5/TABLES/2>

Sioutas, S., Hoffmann, R.W., Pfeil‐Putzien, C., Fischer‐Scherl, T., 1991. Carp Erythrodermatitis (CE) due to an *Aeromonas hydrophila* Infection. J. Vet. Med. Ser. B 38, 186–194. <https://doi.org/10.1111/J.1439-0450.1991.TB00860.X>

Sirri, R., Bianco, C., Zuccaro, G., Turba, M.E., Mandrioli, L., 2016. Hernia of the swim bladder (aerocystocele) with concurrent mycotic granulomatous inflammation and swim bladder carcinoma in a wild mullet (*Mugil cephalus*). J. Vet. Diagnostic Investig. 28, 739–743. <https://doi.org/10.1177/1040638716663600>

Sirri, R., Mandrioli, L., Zamparo, S., Errani, F., Volpe, E., Tura, G., Barbé, T., Ciulli, S., 2020. Swim Bladder Disorders in Koi Carp (*Cyprinus carpio*). Animals 10, 1974. <https://doi.org/10.3390/ANI10111974>

Tung, C.W., Wang, C.S., Chen, S.N., 1999. Histological and electron microscopic study on Macrobrachium muscle virus (MMV) infection in the giant freshwater prawn, *Macrobrachium rosenbergii* (. de Man), cultured in Taiwan. J. Fish Dis. 22, 319–323. <https://doi.org/10.1046/J.1365-2761.1999.00172.X>

Valladão, G.M.R., Alves, L.O., Pilarski, F., 2016. Trichodiniasis in Nile tilapia hatcheries: Diagnosis, parasite: host-stage relationship and treatment. Aquaculture 451, 444–450. <https://doi.org/10.1016/J.AQUACULTURE.2015.09.030>

Vilela, R., Taylor, J.W., Walker, E.D., Mendoza, L., 2015. *Lagenidium giganteum* Pathogenicity in Mammals. Emerg. Infect. Dis. 21, 290–297. <https://doi.org/10.3201/EID2102.141091>

Vincent, A.G., Lotz, J.M., 2007. Advances in Research of Necrotizing Hepatopancreatitis Bacterium (NHPB) Affecting Penaeid Shrimp Aquaculture. Rev. Fish. Sci. 15, 63–73. <https://doi.org/10.1080/10641260601079902>

Yazid, S.H.M., Daud, H.M., Azmai, M.N.A., Mohamad, N., Nor, N.M., 2021. Estimating the Economic Loss Due to Vibriosis in Net-Cage Cultured Asian Seabass (*Lates calcarifer*): Evidence from the East Coast of Peninsular Malaysia. Front. Vet. Sci. 8, 644009. <https://doi.org/10.3389/FVETS.2021.644009/BIBTEX>

Zeng, W., Wang, Q., Wang, Y., Zhao, C., Li, Y., Shi, C., Wu, S., Song, X., Huang, Q., Li, S., 2016. Immunogenicity of a cell culture-derived inactivated vaccine against a common virulent isolate of grass carp reovirus. Fish Shellfish Immunol. 54, 473–480. <https://doi.org/10.1016/J.FSI.2016.04.133>
